# Respiratory and oxygen saturation data for improved modelling and detection of obstructive sleep apnoea

**DOI:** 10.1016/j.dib.2026.112711

**Published:** 2026-03-24

**Authors:** Jordan F. Hill, Ella F.S. Guy, Jaimey A. Clifton, Christopher G. Pretty, J. Geoffrey Chase

**Affiliations:** Department of Mechanical Engineering, University of Canterbury, Christchurch 8041, New Zealand

**Keywords:** Apnoea detection, Pulse oximetry, Airflow resistance, Pulmonary mechanics, Physiological modelling

## Abstract

Twenty healthy adults (10 male, 10 female) participated in a data collection trial designed to capture arterial and venous pulsatile waveforms for oxygen saturation estimation, along with respiratory pressure and flow data to support modelling and prediction of obstructive sleep apnoea (OSA) events. Each participant completed eight one-minute tests while wearing a full-face mask connected to a custom inline pressure and flow sensor, with a Fisher & Paykel SleepStyle CPAP device setting postive PEEP levels at 0, 4, and 8 cmH₂O. A custom reflectance pulse oximeter was taped to the right side of the neck to capture pulsatile waveforms from the carotid artery and internal jugular vein, while a commercial BTIMOS sat801+ pulse oximeter recorded arterial oxygen saturation (SpaO₂) and heart rate from the right index finger. Test conditions included baseline breathing and simulated apnoeas involving 10- and 20-second breath-holds at each PEEP level. Ethical approval was granted by the University of Canterbury Human Research Ethics Committee (Ref: HREC 2024/163/LR-PS). This data aims to support improved apnoea detection, enabling better adaptive control of positive airway pressure (PAP) therapy based on oxygen saturation and respiratory signals.

Specifications Table

**Table:** 

Subject	Engineering & Materials science
Specific subject area	Respiratory and Pulse Oximetry dataset to support physiological modelling for obstructive sleep apnoea assessment
Type of data	Photodiode voltage raw data is in .csv PQ Processed data is in .csv (venturi pressures) Demographic data is in a .csv file Example figures are in .png format Code files are included in .mat format
Data collection	A custom inline pressure and flow meter, a custom reflectance pulse oximeter, and a commercial BTIMOS sat801+ pulse oximeter were used to collect respiratory and oxygen saturation data from 20 adult participants (10 male, 10 female) during eight one-minute trials. Each participant wore a full-face mask with an inline filter connected in series to the pressure and flow meter. The custom pulse oximeter was positioned on the right side of the neck over the carotid artery and internal jugular vein, while the commercial device was placed on the right index finger. Trials consisted of baseline breathing and simulated apnea events involving 10- and 20-second breath-holds at positive end-expiratory pressure levels of 0, 4, and 8 cmH₂O set by a Fisher & Paykel SleepStyle CPAP device.
Data source location	Low Risk Clinical Unit Mechanical Engineering Department University of Canterbury Christchurch New Zealand
Data accessibility	Repository name: Respiratory and Pulse Oximetry Waveforms from Healthy Adults During Simulated Apnoea Events Data identification number: RRID:SCR_007345 Direct URL to data: https://doi.org/10.13026/60xk-ky06

## 1 Value of the Data

• These data combine respiratory pressure-flow measurements with arterial and venous-influenced pulse oximetry signals, enabling detailed analysis of airflow restriction, oxygen extraction, and oxygen desaturation during simulated hypopneas and apneas.
• The dataset includes baseline breathing and breath-hold trials across three PEEP levels (0, 4, and 8 cmH₂O), providing insight into how varying airway support influences flow dynamics, pressure response, and oxygen extraction and saturation trends.
• Researchers can reuse these synchronised multimodal signals for respiratory modelling, simulation, and algorithm development, including integration with other sensors to support personalised, model-based respiratory monitoring approaches. The dataset includes a balanced representation of male and female participants, supporting investigation of potential sex-related differences in respiratory and oxygenation responses, which remain underrepresented in many physiological datasets.
• The dataset is fully open-access and accompanied by signal processing code and device documentation to facilitate reuse, allowing researchers to apply their analyses, incorporate the data into larger studies, or adapt the hardware and software for diverse respiratory monitoring applications.

## 2 Background

Obstructive sleep apnoea (OSA) is a common sleep-related breathing disorder caused by recurrent upper airway collapse, leading to partial (hypopneas) or complete (apnoeas) airflow restriction [[1], [2], [3]]. These episodes are quantified using the apnoea–hypopnoea index (AHI), which guides diagnosis and treatment decisions [2]. Positive airway pressure (PAP) therapy is the standard treatment, delivering continuous or variable pressure to splint open the airway during sleep [[3], [4], [5]]. While effective, current auto-titration PAP devices primarily rely on AHI-based algorithms, which respond to clearly defined apnoeas but may not detect or adjust for earlier or milder respiratory abnormalities, such as hypopneas, oxygen desaturations, or increased airway resistance, where women in particular can present differently than men with such milder apnea events [[6], [7], [8], [9], [10], [11]].

Clinical diagnosis of OSA typically requires overnight polysomnography, which is resource-intensive and time-consuming, limiting accessibility and delaying treatment [12]. To support the development of improved, lower-burden screening and monitoring tools, this dataset was generated using healthy volunteers performing controlled breathing tasks, including breath-holds to simulate apnoeas, at varying levels of positive end-expiratory pressure (PEEP). This dataset provides respiratory and oximetry waveforms under defined, repeatable conditions without relying on patients diagnosed with OSA. The dataset is intended to support the development of models and signal processing tools which could facilitate simpler, more personalised OSA screening and care outside traditional clinical environments.

## 3 Data Description

**Fig. 1:**
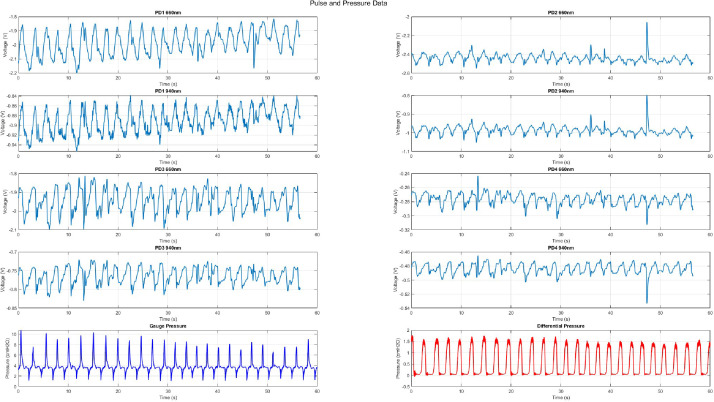
Example figure produced from plotting code showing normal breathing at 4cmH2O. Top eight graphs are neck pulse waveforms for photodiodes (PD) 1–4 for both 660 nm and 940 nm. The bottom two are from the airway sensor showing gauge and differential pressure.

**Fig. 2:**
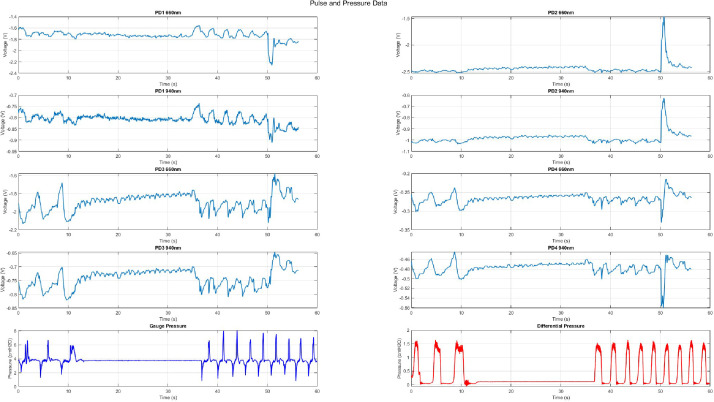
Example figure produced from plotting code showing 20 s breath hold at 4cmH2O. Top eight graphs show the change in neck pulse waveform changing from respiratory modulation to venous pulse during the breath hold, corresponding with the airway sensor detected breath holds.

The data collected is outlined in Table 1 by data type, folder, and filename/format. Files are saved for each subject (1 to 20). For simplicity Subject 1 has been shown in all filenames. Replace the 1 with the subject number (Figs. 1 and 2).

**Table 1:** Data description as displayed on PhysioNet.

Data Type	Folder	File names(s)/format(s)	Data
Raw Pulse Data	Neck_Pulse_ Oximeter_Data / Subject1	*‘Subject1_Baseline.csv’* *‘Subject1_0cmH2O_normal_pulse.csv’* *‘Subject1_4cmH2O_normal_pulse.csv’* *‘Subject1_4cmH2O_apnea1_pulse.csv’* *‘Subject1_4cmH2O_apnea2_pulse.csv’* *‘Subject1_8cmH2O_normal_pulse.csv’* *‘Subject1_8cmH2O_apnea1_pulse.csv’* *‘Subject1_8cmH2O_apnea2_pulse.csv’*	-Time in [s] -PD1-PD4 are photodiode voltages for 660 nm wavelength -PD1_9-PD4_9 are photodiode voltages for 940 nm wavelength
Processed PQ Data	Inline_PQ_Data / Subject1	*Subject1_0cmH2O_normal.csv’* *‘Subject1_4cmH2O_normal.csv’* *‘Subject1_4cmH2O_apnea1.csv’* *‘Subject1_4cmH2O_apnea2.csv’* *‘Subject1_8cmH2O_normal.csv’* *‘Subject1_8cmH2O_apnea1csv’* *‘Subject1_8cmH2O_apnea2.csv’*	-Time [s] -Gauge Pressure [cmH2O] -Inspiratory differential pressure [cmH2O]
Demographic data	Base	*‘Demographic_Data.csv’*	-Subject Number -Sex [M/F] -Height[cm] -Weight [kg] -Age [years] -Neck Circumference [cm] -BMI -Fitness Level -Average Trial Arterial SpaO_2_ [%] -Average Trial Heart Rate [bpm] -History of asthma -History of smoking -History of vaping
Code	Code	*‘DataCollection_InlineSensor.m’* *‘Plotting_Code.m’* *‘natsort.m’* *‘*Fig. 1*.jpg’* *‘*Fig. 2*.jpg’*	-Pressure and flow device data collection code in MATLAB -Plotting figure generation code -Sorter for files in chronological order -Example figures which are produced in the plotting code

## 4 Experimental Design, Materials and Methods

A custom inline sensor measured gauge pressure and inspiratory differential pressure at the airway, from which flow and tidal volume were derived. The sensor was a simple inline Venturi connected in series with a filter and a full-face mask. The venturi inlet was then attached to a CPAP device (SleepStyle SPSCAA, Fisher and Paykel Healthcare, Auckland, New Zealand) to provide PEEP. A transmission finger pulse oximeter (Bitmos SAT801+, Düsseldorf, Germany) was placed on the right index finger and recorded continuously throughout the trial. This device provided stable heart rate and arterial oxygen saturation (SpaO_2_) measurements, which remained relatively constant during the tests, with no desaturation events detected from the finger sensor for any subject. Averages of heart rate and SpaO_2_ were taken and can be found in the demographic data.

A reflectance pulse oximeter was used to detect waveforms from the carotid artery and internal jugular vein (IJV). The device was lightly taped to the right-hand side of the neck to avoid applying pressure after the locations of the carotid artery and IJV were identified using ultrasound imaging. The sensor consisted of eight LEDs emitting at 660 nm and 940 nm wavelengths, and four photodiodes which detected reflected light signals. Each photodiode recorded distinct signals representing either venous, arterial, or mixed blood sources, with some tests also capturing breathing-related waveforms. These signals were processed to extract estimated SpaO_2_, venous oxygen saturation (SpvO_2_), oxygen extraction ratio (O_2_ER), heart rate, and respiratory rate. All measurements were taken simultaneously, enabling direct comparison across modalities.

Demographic data were collected via questionnaire and physical measurements for weight, height, and neck circumference. This information was used to assess variability in respiratory signals relative to factors such as body index, neck circumference, smoking status, asthma, vaping habits, and fitness level, which can influence both respiratory mechanics and oximetry data. Additionally, these demographic variables provide context for comparison with other studies.

Each subject completed eight tests in total. Each test was recorded and saved as a separate file. Initially, a baseline normal breathing at zero PEEP (ZEEP, 0 cmH₂O) for 60 s was conducted. The PEEP was then increased to 4 cmH₂O, and normal breathing occurred for another 60 s. Subjects then performed three breaths followed by a self-timed 10-second breath hold at 4 cmH₂O PEEP, after which they resumed normal breathing for the remainder of the 60-second trial period. Subjects were provided with a stopwatch for more accurate timing of their breath holds. This trial was then repeated with a 20-second breath hold. The same breath and breath-hold protocol was repeated at 8 cmH₂O PEEP. Simulated apnoea events were included to investigate whether respiratory and oximetry signals could identify precursors leading up to hypopnoea, apnoea, and oxygen desaturation. All data collection and processing were conducted using MATLAB (Matlab 2024b, The Mathworks Inc, Natick, MA, USA), with example figures demonstrating the potential analyses included alongside the dataset. Data collection and processing software is included in the dataset.

## Limitations

Data was collected from only 20 healthy adults simulating OSA events, rather than from patients with diagnosed OSA. Oxygen saturation was measured using a custom, uncalibrated reflectance pulse oximeter placed on the neck and compared to a commercial transmittance pulse oximeter on the finger. Venous oxygen saturation and oxygen extraction ratios cannot be precisely determined without invasive blood gas sampling, which was not performed. Therefore, arterial oxygen saturation comparisons are more reliable than venous estimates.

Signal quality from the neck sensor was occasionally degraded due to subject movement or irregular breathing patterns. The mixing of arterial and venous signals further reduced clarity. However, filtering mitigated most of these effects. Differences in individual breathing patterns also led to inconsistent presence of respiratory waveforms in the pulse oximetry data from the neck sensor.

Tidal volume estimation may have been impacted by leakage inherent to full-face masks, along with possible system leaks or sensor limitations. Additionally, data collected at ZEEP showed poor quality due to the expected very low airflow. Future work could mitigate these limitations by including a clinical OSA cohort with associated ethics approval, implementing invasive reference measurements, and/or refining hardware and interface designs.

## Ethics Statement

Ethical consent for the trial was granted by the Human Research Ethics Committee at the University of Canterbury (Ref: HREC 2024/163/LR-PS). Subjects gave written consent before the trial after both written and verbal explanations of the procedure. Subjects consented to the publication of their de-identified data.

## CRediT Author Statement

**Jordan F. Hill**: Conceptualisation, Methodology, Software, Validation, Investigation, Data curation, Writing - original draft, Visualisation. **Ella F. S. Guy**: Conceptualisation, Methodology, Software, Validation, Investigation, Data curation, Writing - review & editing, Visualisation. **Jaimey A. Clifton:** Conceptualisation, Investigation. **J. Geoffrey Chase:** Conceptualisation, Methodology, Resources, Writing - review & editing, Supervision, Funding acquisition.

## Data Availability

(PhysioNet)Respiratory and Pulse Oximetry Waveforms from Healthy Adults During Simulated Apnoea Events (Original data).
